# Supplementary material for: A Systems Analysis of Phenotype Heterogeneity in APOE*3Leiden.CETP Mice Induced by Long-Term High-Fat High-Cholesterol Diet Feeding
Source: Nutrients. 2022 Nov 21;14(22):4936. doi: 10.3390/nu14224936 (PMC9698005; doi:10.3390/nu14224936)
Supplement: Supplementary file 1 [file nutrients-14-04936-s001.zip › nutrients-1968751-supplementary.pdf]

Suppl. S1: Finding appropriate settings within the ADAPT procedure for the model

The computational approach of ADAPT has been described previously (1). The method makes use of the fact that most physiological processes change relatively slowly over time, and that taking this into account in a computational model makes parameter estimation more accurate. Since the efficiency of the ADAPT procedure on achieving a good fit to the data is in part dependent on the regularization imposed by the λ parameter, we first tested whether for this model size and these number of constraints, a λ of 0.01 was appropriate. We therefore varied λ between 10^{-5} and 10^{5} and assessed the goodness-of-fit based on the sum of squares of the residual error. For every lambda, we repeated the procedure for 100 iterations. Every iteration used a different set of data splines, and the set of initial parameter values for every iteration was taken from a uniform distribution of the log-transformed linear space between 10^{4} and 10^{-4}. Between λs however, iterations made use of the same data splines and set of initial parameter values, so that the effect of varying λ could be isolated. Iterations for which the optimizer failed to converge for one or multiple λ values were discarded. We then found that a λ value of 0.01 is indeed appropriate (**Figure S1**). To find out what number of time steps was appropriate, we used a λ of 0.01 and ran ADAPT for 100 iterations while varying the number of time steps between 2 and 1000. We then found that 200 time steps were appropriate (**Figure S2**). This set of initial parameters was then used for subsequent runs on the full time span, using a λ of 0.01 and 200 time steps. Finally, of results obtained in this way, the best 10% of a 1000 fits are displayed, unless stated otherwise.

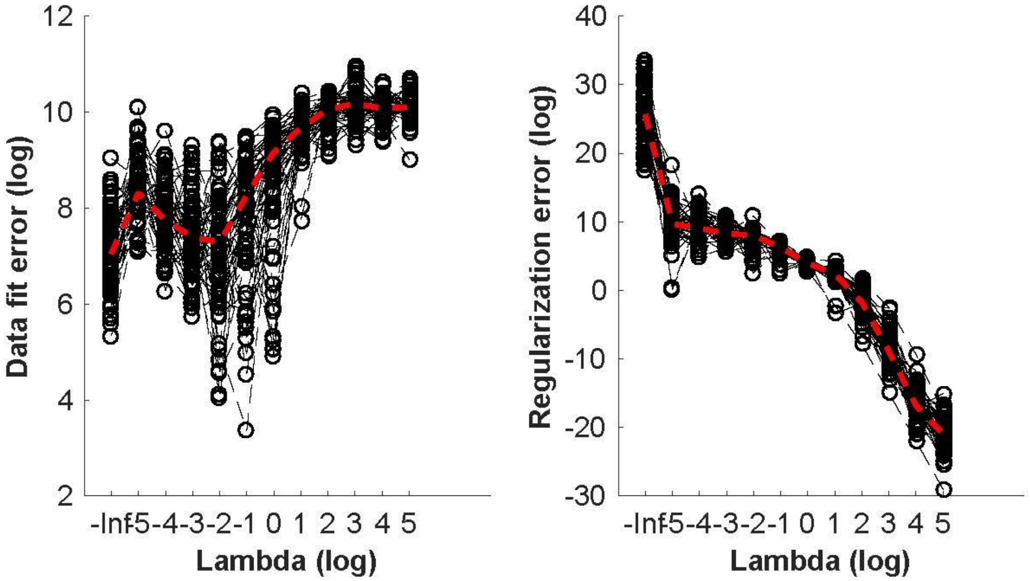

Figure S1

The data fit error and regularization error for different lambdas (logarithmic scale). Note that for lambda 10^-2 , the data fit error is small and the regularization error has dropped as well, while at larger lambdas decreasing regularization error occurs at the expense of the data fit.

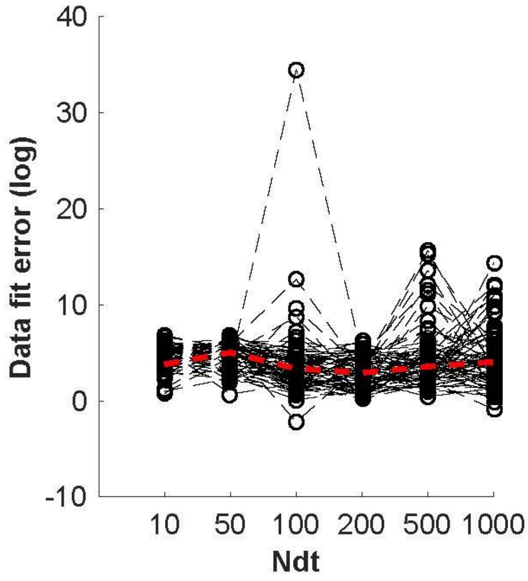

Figure S2

The Data fit error and regularization error for different amount of time steps. Note that there is little difference in data fit error for the different amount of time steps, while beyond 200 time steps the regularization error is not greatly decreased.

1. Tiemann, C. A., J. Vanlier, M. H. Oosterveer, A. K. Groen, P. A. J. Hilbers, and N. A. W. van Riel. 2013. Parameter trajectory analysis to identify treatment effects of pharmacological interventions. *PLoS Comput. Biol.* **9**: e1003166.

Suppl. S2: Translation of Experimental Data to Modeling Constraints

Experimental data were used as constraints for the model in the following ways: The amount of peripheral fat was estimated by assuming a steady lean mass of 24 gram. The peripheral fat, expressed as µmol TG, was then approximated as, (BW-24)*850. To account for de novo lipogenesis, we assumed that the rate of de novo lipogenesis was equal to the amount of newly synthesized fatty acids calculated from the mass-isotopomer-distribution analysis during the labeling period. We further assumed that the composition of fatty acids within the liver did not change over time. Then, we determined the amount of newly synthesized triglyceride by taking the total of palmitate, oleate and stearate produced multiplied by 0.33, 0.62, and 0.05 respectively, according to the respective abundance of these fatty acids in the liver (1). Chain elongation was counted as $1/9^{th}$ of a fatty acid newly synthesized. To account for the pools of plasma volume being dependent on the weight of the animals, we multiplied the measured concentration in the plasma with the theoretical plasma volume as derived from linear least-squares regression of plasma volume against body weight for obese mice, using the data from Yen et al. (1970) (2). We then arrive at the following relation : Plasma Volume = 0.0117 + 0.7704*BW. This relation was used to calculate VLDL-TG production as well. The lumenal content for cholesterol was constrained on the assumption that a mouse has 1 gram of feces. This was done so that the model would not inadvertently accumulate lumenal content, which would be unrealistic.

1. Oosterveer, M. H., T. H. van Dijk, U. J. F. Tietge, T. Boer, R. Havinga, F. Stellaard, A. K. Groen, F. Kuipers, and D.-J. J. Reijngoud. 2009. High fat feeding induces hepatic fatty acid elongation in mice. *PLoS One*. **4**: e6066.

2. Yen, T., J. Stienmetz, and P. J. Simpson. 1970. Blood Volume of Obese (ob/ob) and Diabetic (db/db) Mice (34462). *P.S.E.B.M.* **133**: 307–308.

Suppl. S3: Model Description

Table S1: Description of Model States

Symbol	Description
Lum_TG	Triglyceride in intestinal lumen
Lum_C	Cholesterol in intestinal lumen
Lum_BA	Bile acids in intestinal lumen
Pl_Gluc	Plasma glucose
Pl_FFA	Plasma free fatty acids
Pl_VLDL_TG	Plasma VLDL-TG
Pl_VLDL_C	Plasma VLDL-C
Pl_HDL_C	Plasma HDL-C
Hep_G6P	Hepatic glucose-6-phosphate
Hep_AcoA	Hepatic acetyl-CoA
Hep_TG	Hepatic triglycerides
Hep_FC	Hepatic free cholesterol
Hep_CE	Hepatic cholesterol ester
Hep_BA	Hepatic bile acids
Per_G6P	Peripheral glucose-6-phosphate
Per_AcoA	Peripheral acetyl-CoA
Per_TG	Peripheral triglycerides
Per_C	Peripheral cholesterol

Table S2: Description of Model Parameters

Symbol	Description
gluc_abs	Intestinal glucose absorption
glut_2	Hepatic glucose absorption
glut_134	Peripheral glucose absorption
hep_PFK	Hepatic phosphofructokinase
per_PFK	Peripheral phosphofructokinase
hep_AA	Hepatic amino acids
per_AA	Peripheral amino acids
g6pase	Glucose-6-phosphatase
fat_intake	Dietary fat Intake
hep_chyl_upt	Hepatic fat uptake
per_chyl_upt	Peripheral fat uptake
hep_LDLRf	Hepatic LDLR
per_LDLRf	Peripheral LDLR
hep_CPT1	Hepatic fat oxidation
per_CPT1	Peripheral fat oxidation
hep_CS	Hepatic Krebs cycle
per_CS	Peripheral Krebs cycle
hep_ACC	Hepatic Acetyl CoA Carboxylase
per_ACC	Peripheral Acetyl CoA Carboxylase

**hep_apob**	VLDL-C production
**LPL**	Lipoprotein Lipase
**HSL_ATGL**	Peripheral lipolysis
**CD36**	Hepatic free fatty acid uptake
**chol_intake**	Dietary cholesterol intake
**NPC1L1**	Intestinal cholesterol Uptake
**hep_HMGCR**	Hepatic cholesterol synthesis
**hep_ACAT**	Hepatic cholester esterification
**hep_CEH**	Hepatic cholesterol ester hydrolyzation
**per_HMGCR**	Peripheral cholesterol synthesis
**CYP7A1**	Bile acid synthesis
**ABCA1**	HDL synthesis
**SRB1**	HDL uptake
**PLTP**	VLDL-TG production
**CETP**	Cholesterol Ester Transfer Protein
**pTICE**	Transintestinal cholesterol excretion
**BSEP**	Biliary bile acid secretion
**ABCG5**	Biliary free cholesterol excretion
**k_fec_exc**	Fecal excretion rate
**k_reabsorb**	Biliary reabsorption

Table S3: Description of Model Fluxes

Legend	Symbol	Description	Rate Equation
**j1**	Gluc_abs	Glucose Absorption	gluc_abs
**j2**	Hep_Gluc_upt	Hepatic Glucose Uptake	glut2 * Pl_Gluc
**j3**	Per_Gluc_upt	Peripheral Glucose Uptake	glut134 * Pl_Gluc
**j4**	Hep_glyc	Hepatic Glycolysis	hep_PFK * Hep_G6P
**j5**	Per_Glyc	Peripheral Glycolysis	per_PFK * Per_G6P
**j6**	Hep_AAglc	Hepatic glucogenic amino acids	0.5 * hep_AA
**j7**	Per_AAglc	Peripheral glucogenic amino acids	0.5 * per_AA
**j8**	Hep_AAket	Hepatic ketogenic Amino acids	0.5 * hep_AA
**j9**	Per_AAket	Peripheral ketogenic Amino acids	0.5 * per_AA
**j10**	G6Pase	Hepatic Glucose-6-Phosphatase	g6pase * Hep_G6P
**j11**	Fat_intake	Fat_intake	fat_intake
**j12**	Hep_chylTG_upt	Hepatic Chylomicron Uptake	hep_chyl_upt * Lum_TG
**j13**	Per_chylTG_upt	Peripheral Chylomicron Uptake	per_chyl_upt * Lum_TG
**j14**	HepTG_ox	Hepatic Fat oxidation	hep_CPT1 * Hep_TG
**j15**	PerTG_ox	Peripheral Fat oxidation	per_CPT1 * Per_TG
**j16**	hepKC	Hepatic Krebs Cycle	hep_CS * Hep_AcoA
**j17**	perKC	Peripheral Krebs Cycle	per_CS * Per_AcoA
**j18**	hepDNL	Hepatic De novo lipogenesis	hep_ACC * Hep_AcoA
**j19**	perDNL	Peripheral De novo lipogenesis	per_ACC * Per_AcoA
**j20**	VLDLTG prod	VLDL TG production	apoB * Hep_TG
**j21**	VLDLTG upt	VLDL TG uptake	LPL * Pl_VLDL_TG
**j21**	Lipolysis	Lipolysis	HSL_ATGL * Per_TG
**j22**	FFA_upt	FFA uptake	CD36 * Pl_FFA

**j24**	Chol_Intk	Dietary Cholesterol Intake	chol_intk
**j25**	Remn_chol_upt	Remnant Cholesterol Uptake	NPC1L1 * Lum_C
**j26**	Hep_cholsynt	Hepatic Cholesterol Synthesis	hep_HMGCR * Hep_AcoA
**j27**	Hep_acat	Hepatic acat activity	hep_ACAT * Hep_FC
**j28**	Hep_ceh	Hepatic CEH activity	hep_CEH * Hep_CE
**j29**	Per_cholsynt	Peripheral Cholesterol Synthesis	per_HMGCR * Per_AcoA
**j30**	BA_synth	Bile Acid Synthesis	CYP7A1 * Hep_FC
**j31**	HDLC_prod	HDLC production	ABCA1 * Per_C
**j32**	HDLC_upt	HDLC uptake	SRB1 * Pl_HDL_C
**j33**	VLDLC_prod	VLDLC production	PLTP * Hep_CE
**j12**	Hep_LDLupt	Hepatic LDL uptake	hep_LDLRf * Pl_VLDL_C
**j13**	Per_LDLupt	Peripheral LDL uptake	per_LDLRf * Pl_VLDL_C
**j34**	CETP	CETP-activity	pCETP * Pl_VLDL_TG
**j35**	TICE	Trans-Intestinal Cholesterol Excretion	pTICE * Pl_VLDL_C
**j36**	BA_sec	Bile Acid Secretion	BSEP * Hep_BA
**j37**	Chol_sec	Cholesterol secretion	ABCG5 * Hep_FC
**j38**	FecTG_exc	Fecal TG excretion	fec_exc * Lum_TG
**j39**	FecC_exc	Fecal Cholesterol excretion	fec_exc * Lum_C
**j40**	FecBA_exc	Fecal BA excretion	fec_exc * Lum_BA
**j41**	FecBA_reabsorb	Fecal BA reabsorption	reabsorb * Lum_BA

Model Equations:

1) dLUM_TG/dt = Fat_Intk - Hep_ChylTG_Upt - Per_ChylTG_Upt - FecTG_exc
2) dLUM_C/dt = Chol_Intk + TICE + Chol_Sec - Remn_Chol_Upt - FecC_exc
3) dLUM_BA/dt = BA_sec - FecBA_reabsorb - FecBA_exc
4) dPl_GLUC/dt = Gluc_abs - Hep_Gluc_Upt - Per_Gluc_Upt + G6pase
5) dPl_FFA/dt = 3*Lipolysis - FFA_upt
6) dPl_VLDL_TG/dt = VLDLTG_prod – VLDLTG_upt
7) dPl_VLDL_C/dt = VLDLC_prod + CETP - hep_LDLupt - per_LDLupt - TICE
8) dPl_HDL_C/dt = HDLC_prod - HDLC_upt - CETP
9) dHep_G6p/dt = Hep_Gluc_Upt + Hep_AAglc - Hep_glyc - G6pase
10) dHep_AcoA/dt = 2*Hep_glyc + 21.4*HepTG_ox + 2*Hep_AAket - HepKC - HepDNL - Hep_cholsynt
11) dHep_TG/dt = Hep_ChylTG_Upt + FFA_upt/3 + HepDNL/21.4 - HepTG_ox - VLDLTG_prod

12) dHep_FC/dt = Remn_Chol_Upt - Chol_Sec + Hep_Cholsynt/13.5 - BA_synt - Hep_acat + Hep_ceh

13) dHep_CE/dt = Hep_LDLupt + HDLC_upt - VLDLC_prod + Hep_acat - Hep_ceh

14) dHep_BA/dt = BA_synt - BA_sec + FecBA_reabsorb

15) dper_G6P/dt = Per_gluc_upt - Per_glyc + Per_AAglc

16) dper_AcoA/dt = 2*Per_glyc + 21.4*PerTG_ox + 2*Per_AAket - PerKC - PerDNL - Per_cholsynt

17) dVLDL_TG_upt/dt = VLDLTG_upt + Per_chylTG_upt + PerDNL/21.4 - Lipolysis - PerTG_ox

18) dVLDL_C_upt/dt = Per_Cholsynt/13.5 + Per_LDLupt - HDLC_prod

Suppl. Figures : Figure S3 – Figure S16

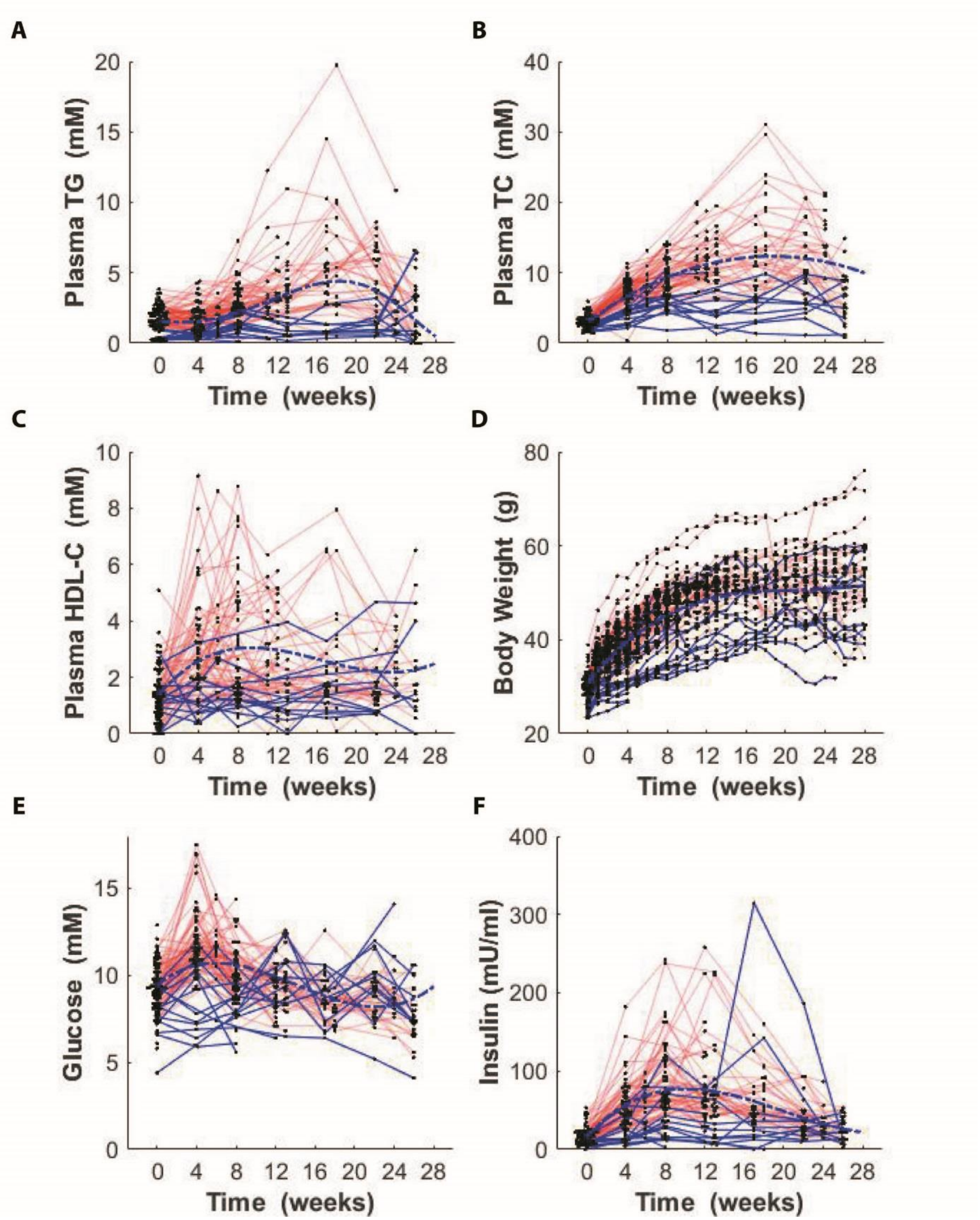

Figure S3

Responders are marked in red and non-responders are marked in blue. Note how low plasma TG in non-responders is accompanied by lower body weights and less insulin resistance.

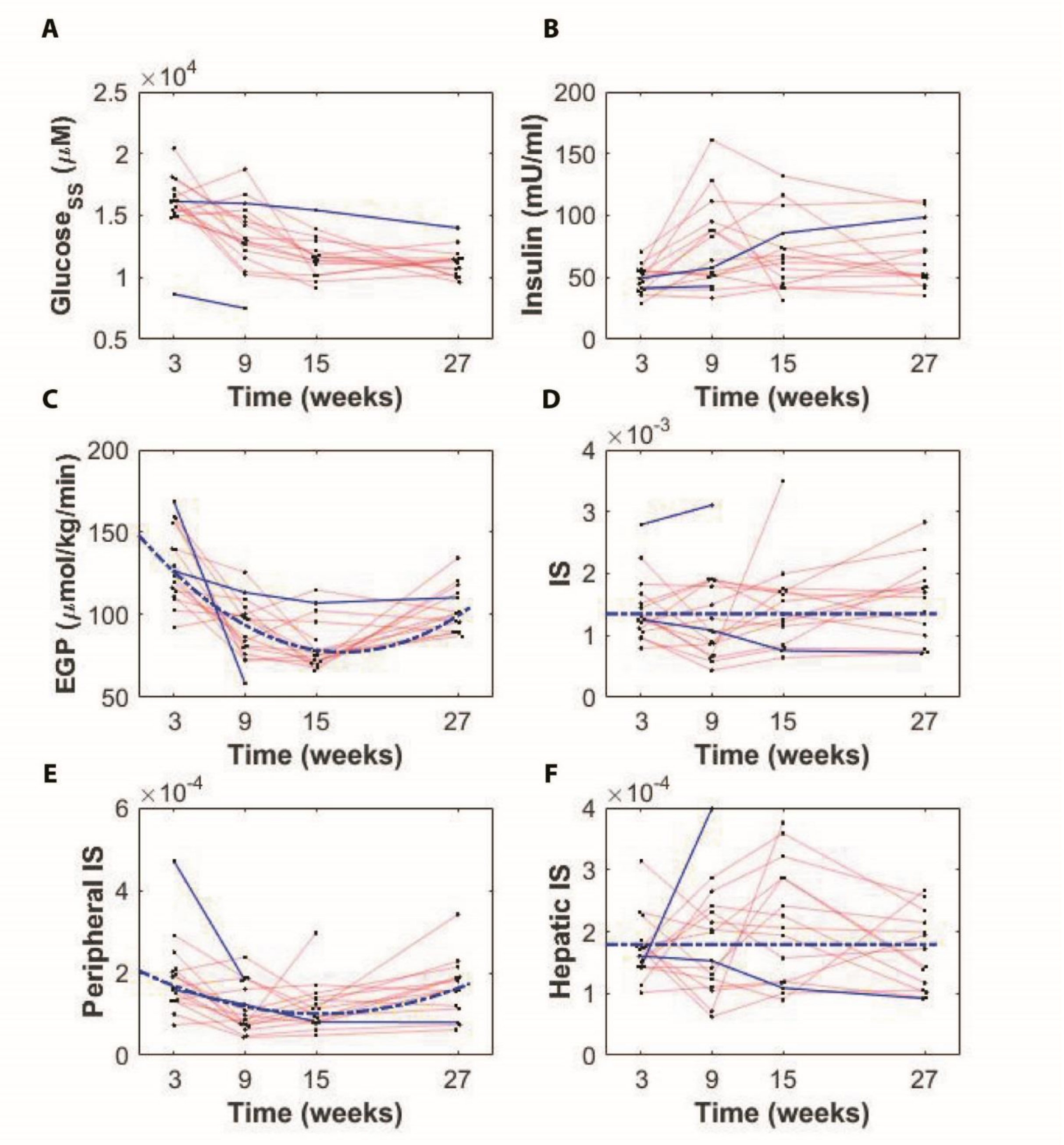

Figure S4

Evolution of insulin sensitivity of responders (red) and non-responders (blue) over the course of the experiment. One of the non-responders had to be terminated early because of a rapid decrease in body weight. The non-responder that had to be terminated early was marked by low insulin-resistance.

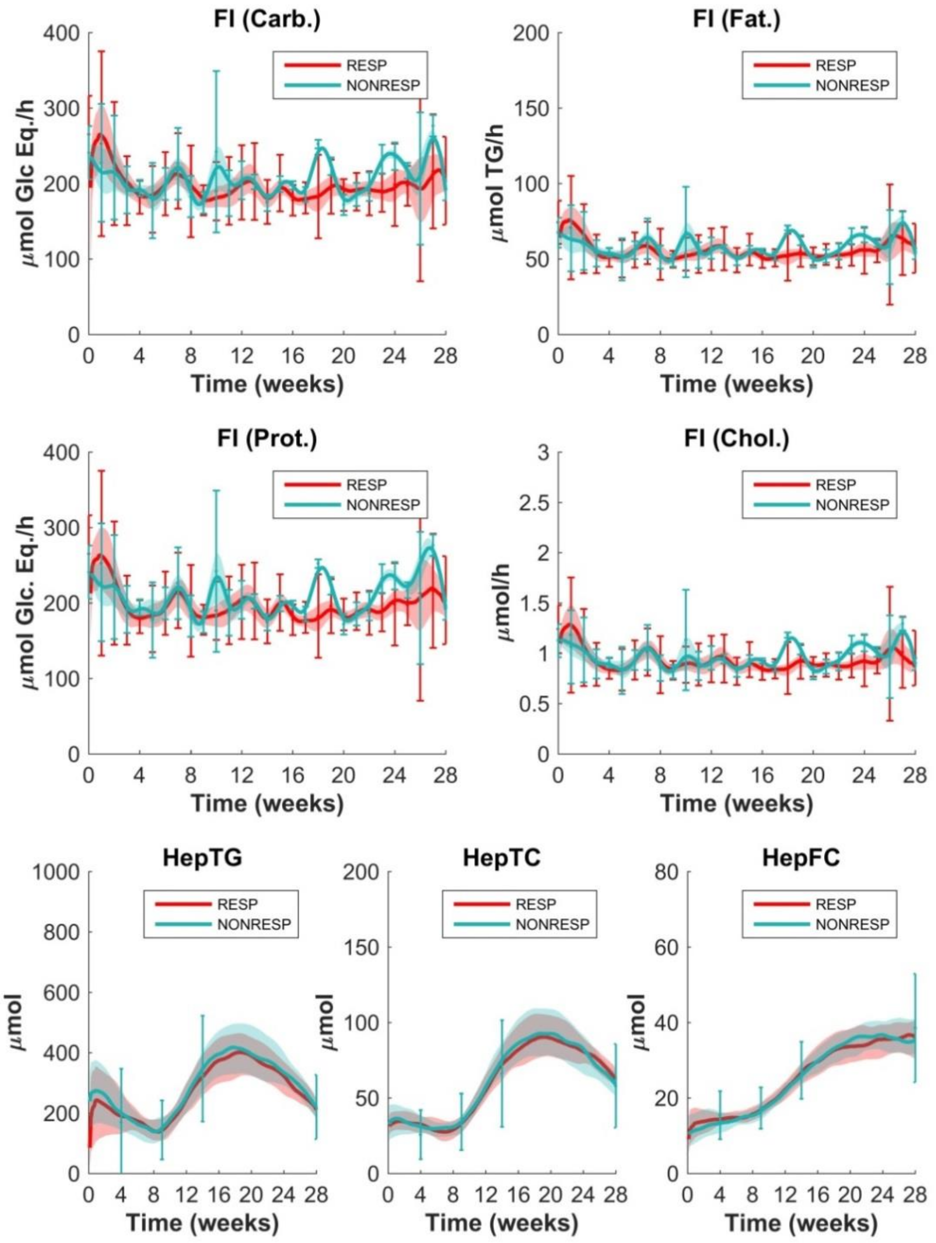

Figure S5

Simulation results for food intake (FI), hepatic TG (HepTG), total cholesterol (HepTC) and free cholesterol (HepFC). The curve represents the median values, whereas the area around the line denotes 30% of solutions around the median. Error bars denote the standard deviation of the experimental data.

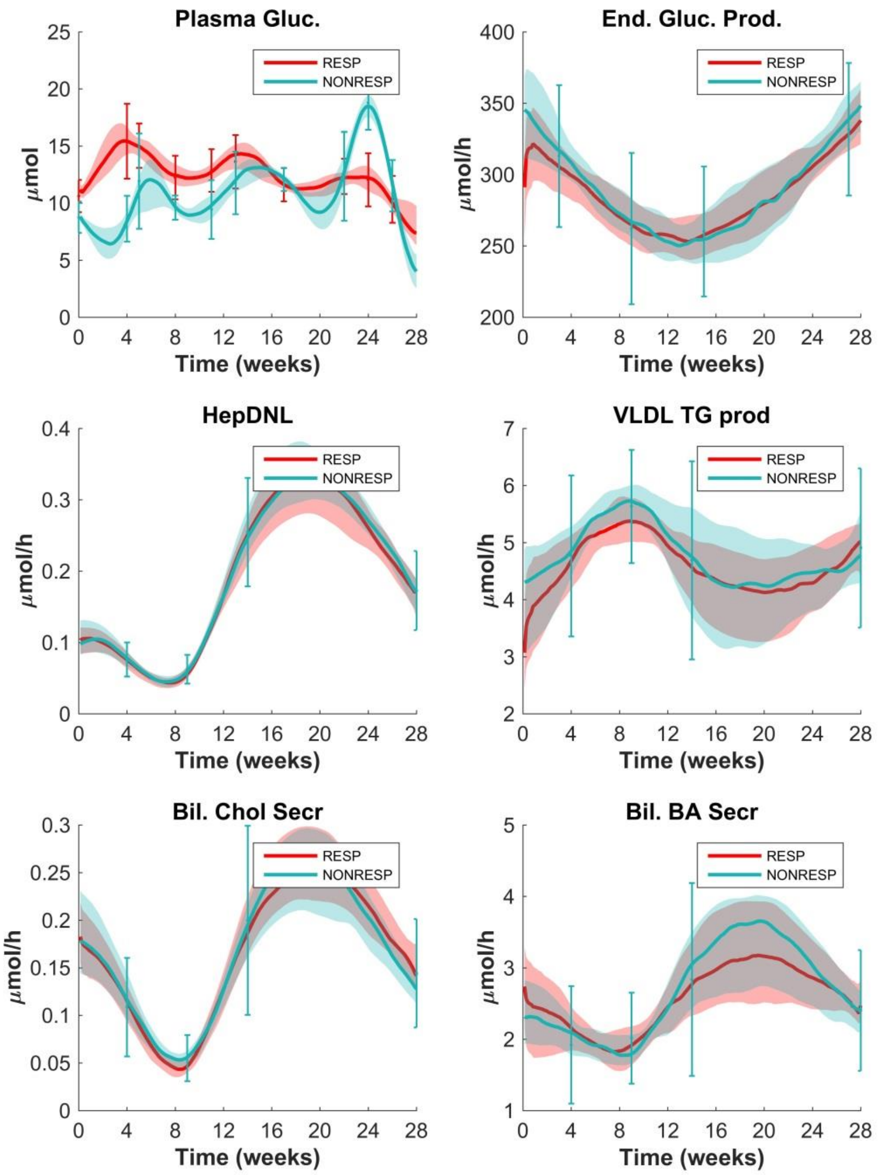

Figure S6

Simulation results for plasma glucose (Plasma Gluc.), endogenous glucose production (End. Gluc. Prod.), hepatic de novo lipogenesis (HepDNL), VLDL-TG production (VLDL TG prod) and biliary secretion of cholesterol (Bil. Chol Secr) and bile acids (Bil. BA Secr). The curve represents the median values, whereas the area around the curve denotes 30% of solutions around the median. Error bars denote the standard deviation of the experimental data.

.

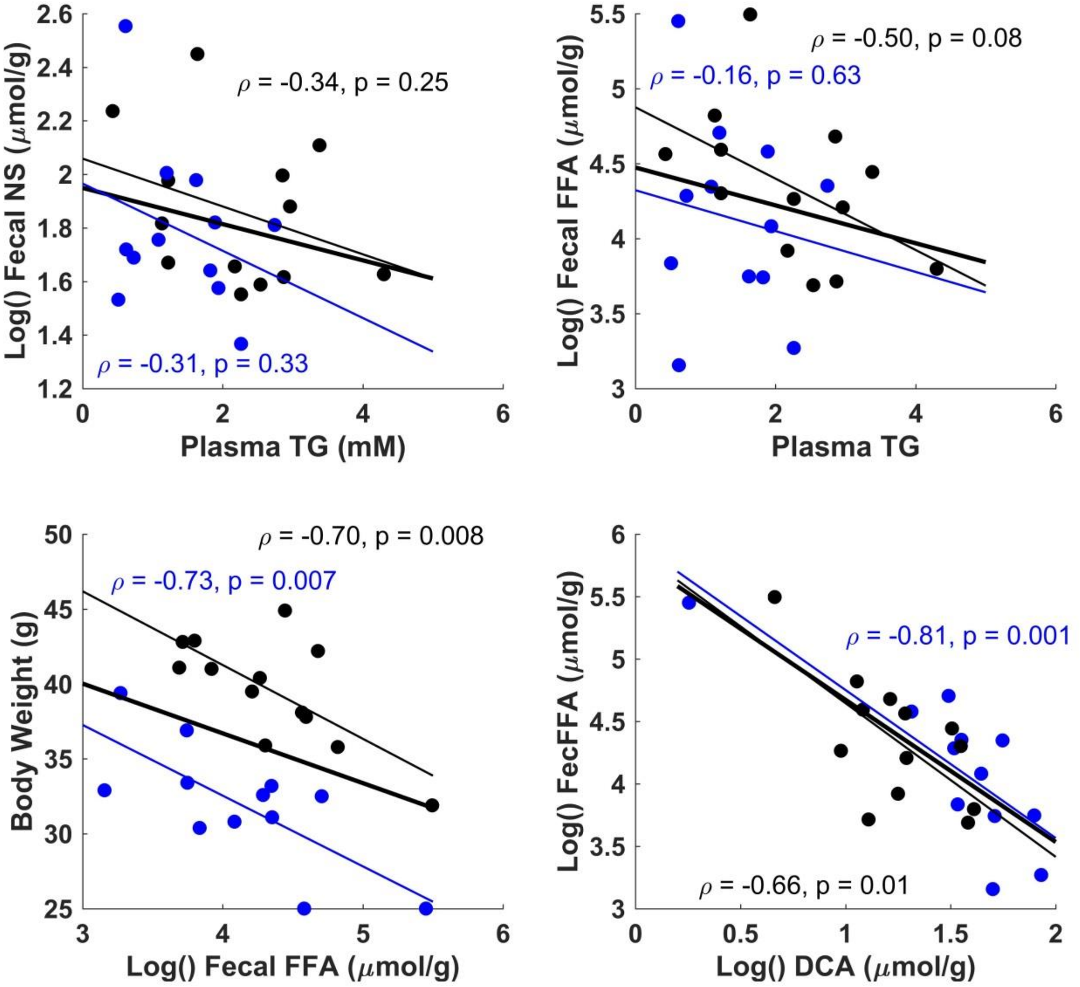

Figure S7

Correlations between plasma TG and fecal neutral sterols (Fecal NS) (A), plasma TG and fecal free fatty acids (FFA) (B), fecal FFA and body weight (C), and the correlation between deoxycholic acid (DCA) and fecal FFA content (D). Note the absence of correlation between plasma TG and fecal neutral sterols and the strong correlation between fecal FFA content and body weight.

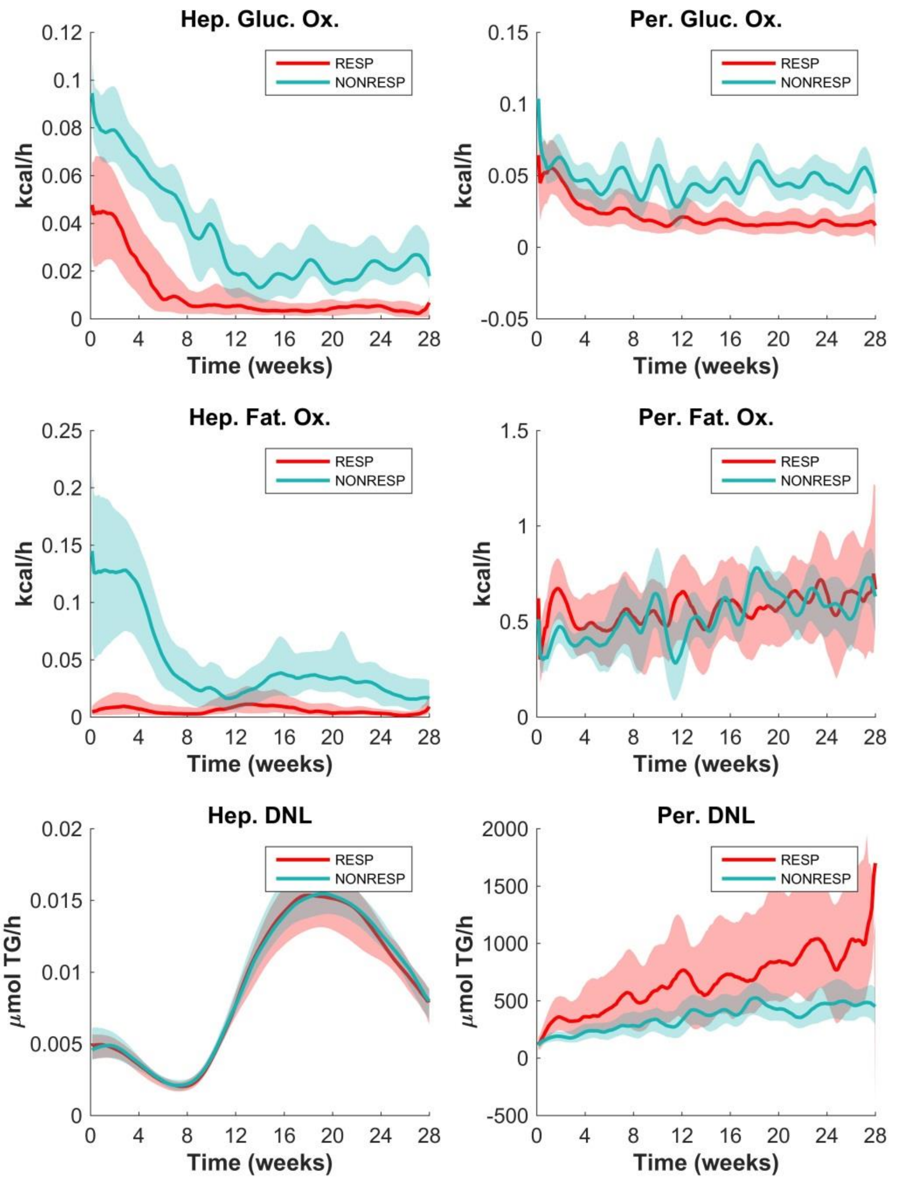

Figure S8

Predictions for hepatic and peripheral glucose oxidation (Hep. Gluc. Ox., Per. Gluc. Ox.), fat oxidation (Hep. Fat. Ox., Per. Fat. Ox.) and de novo lipogenesis (Hep. DNL, Per. DNL). The curve represents the median values, whereas the area around the line denotes 30% of solutions around the median.

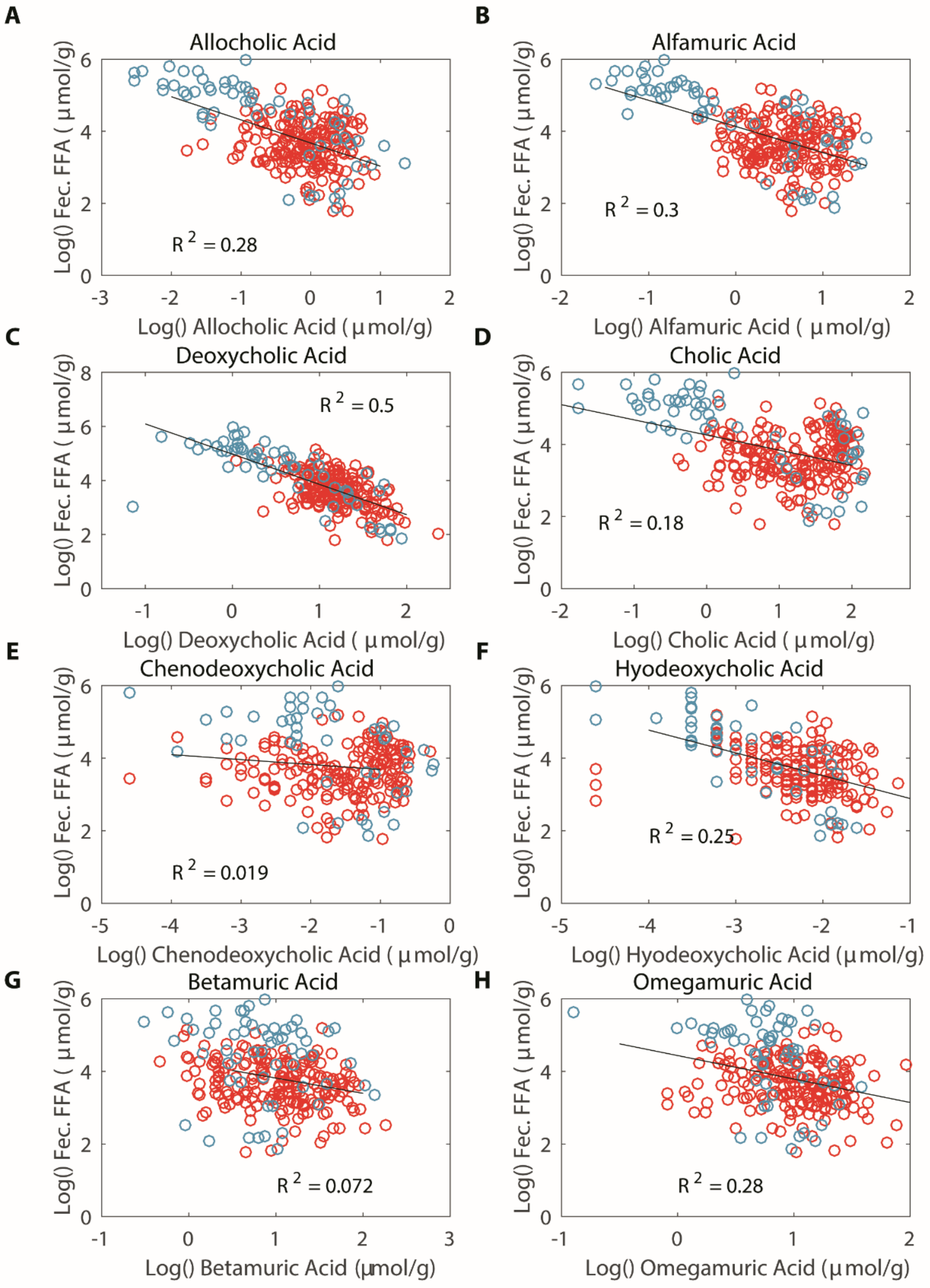
A
Allocholic Acid
Log() Fec. FFA (µmol/g)
R 2 = 0.28
Log() Allocholic Acid (µmol/g)
B
Alfamuric Acid
Log() Fec. FFA (µmol/g)
R 2 = 0.3
Log() Alfamuric Acid (µmol/g)
C
Deoxycholic Acid
Log() Fec. FFA (µmol/g)
R 2 = 0.5
Log() Deoxycholic Acid (µmol/g)
D
Cholic Acid
Log() Fec. FFA (µmol/g)
R 2 = 0.18
Log() Cholic Acid (µmol/g)
E
Chenodeoxycholic Acid
Log() Fec. FFA (µmol/g)
R 2 = 0.019
Log() Chenodeoxycholic Acid (µmol/g)
F
Hyodeoxycholic Acid
Log() Fec. FFA (µmol/g)
R 2 = 0.25
Log() Hyodeoxycholic Acid (µmol/g)
G
Betamuric Acid
Log() Fec. FFA (µmol/g)
R 2 = 0.072
Log() Betamuric Acid (µmol/g)
H
Omegamuric Acid
Log() Fec. FFA (µmol/g)
R 2 = 0.28
Log() Omegamuric Acid (µmol/g)

Figure S9

Correlations between individual bile acid species and fecal FFA concentrations irrespective of time spent on HFCD. Non-responder samples are colored in blue, and responder samples are colored in red. Note that especially the correlation between deoxycholic acid and fecal FFA is strong.

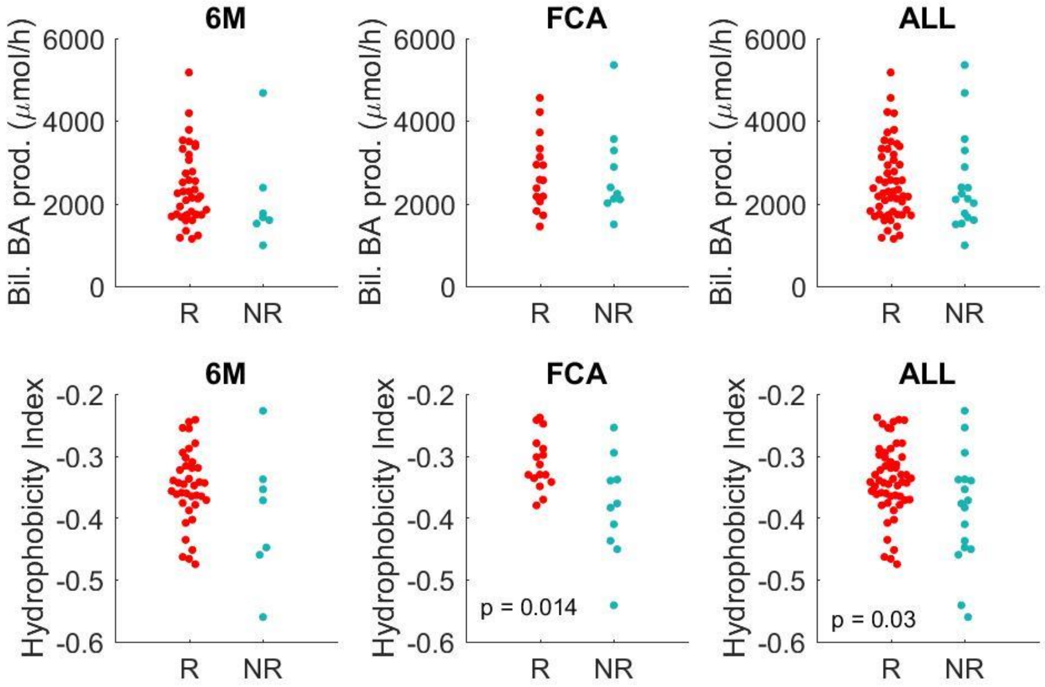

Figure S10

Biliary bile acid secretion rate and hydrohobicity index of biliary bile acids for cohorts followed for up to 6 months (6M), the validation cohorts used for measurement of fractional cholesterol absorption (FCA) and both together (ALL). Note how the hydrophobicity index is lower for non-responders (NR) vs. responders (R). Whenever the difference between R and NR-groups was statistically significant (αϴ.05) using the Wilcoxon rank-sum test, p-values are shown.

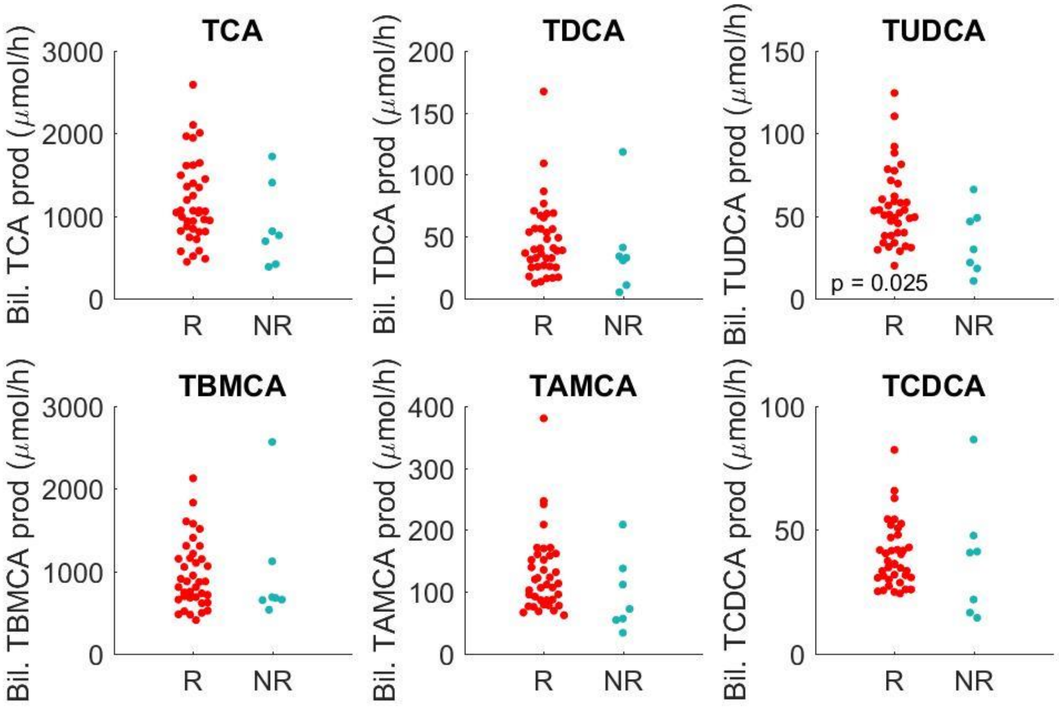

Figure S11

Biliary tauro-cholate (TCA), tauro-deoxycholate (TDCA), tauro-ursodeoxycholate (TUDCA), tauro-beta-muricholate (TBMCA), tauro-alpha-muricholate (TAMCA) and tauro-chenodeoxycholate (TCDCA) for responders (R) and non-responders (NR) of all mice followed for up to 6 months on HFCD. Whenever the difference between R and NR-groups was statistically significant (α=0.05) using the Wilcoxon rank-sum test, p-values are shown.

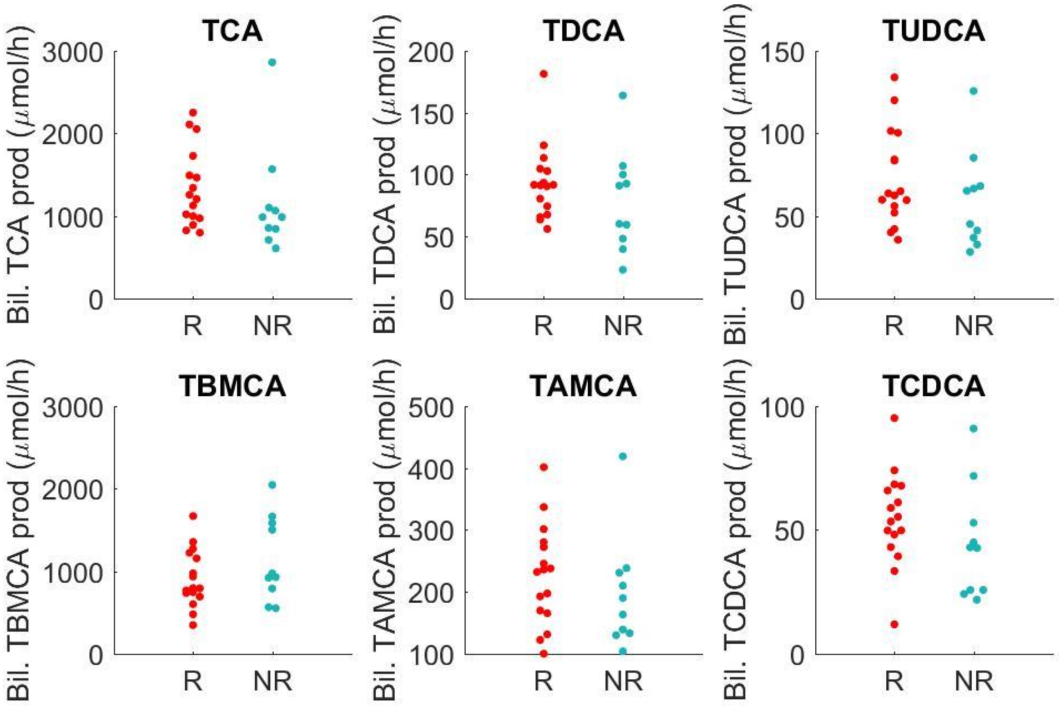

Figure S12

Biliary tauro-cholate (TCA), tauro-deoxycholate (TDCA), tauro-ursodeoxycholate (TUDCA), tauro-beta-muricholate (TBMCA), tauro-alpha-muricholate (TAMCA) and tauro-chenodeoxycholate (TCDCA) for responders (R) and non-responders (NR) of mice on HFCD for 2 months used for measurement of frational cholesterol absorption. Whenever the difference between R and NR-groups was statistically significant ($\alpha = 0.05$) using the Wilcoxon rank-sum test, p-values are shown.

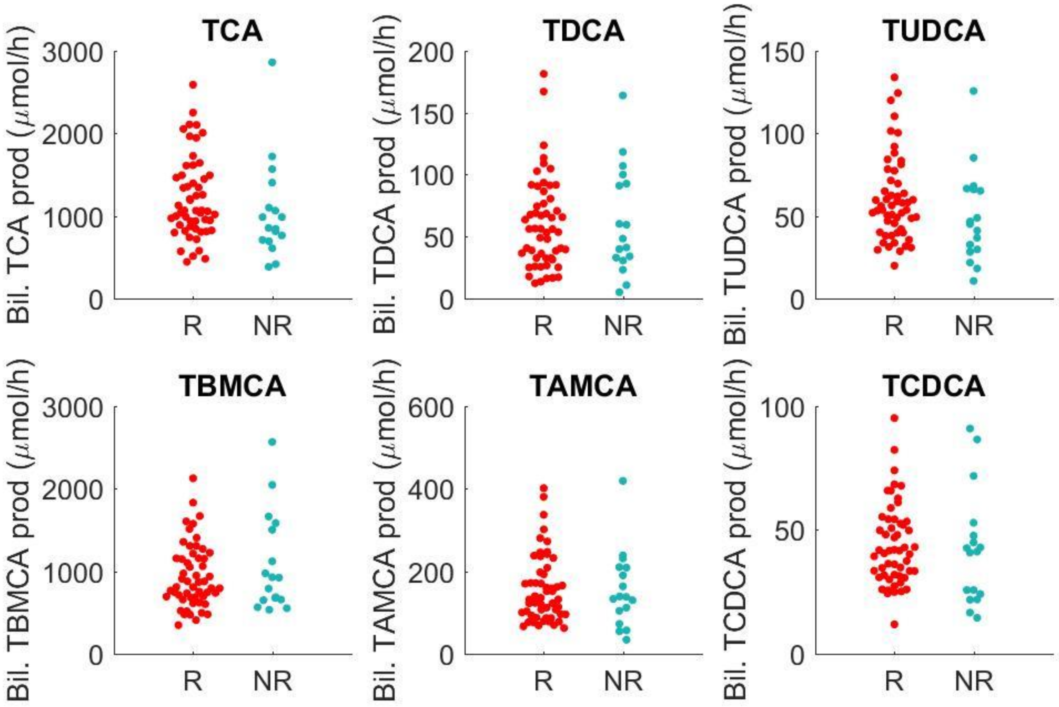

Figure S13

Biliary tauro-cholate (TCA), tauro-deoxycholate (TDCA), tauro-ursodeoxycholate (TUDCA), tauro-beta-muricholate (TBMCA), tauro-alpha-muricholate (TAMCA) and tauro-chenodeoxycholate (TCDCA) for responders (R) and non-responders (NR) of all mice followed for up to 6 months on HFCD and mice used for measurement of fractional cholesterol absorption after 2 months of HFCD together. Whenever the difference between R and NR-groups was statistically significant ($\alpha=0.05$) using the Wilcoxon rank-sum test, p-values are shown.

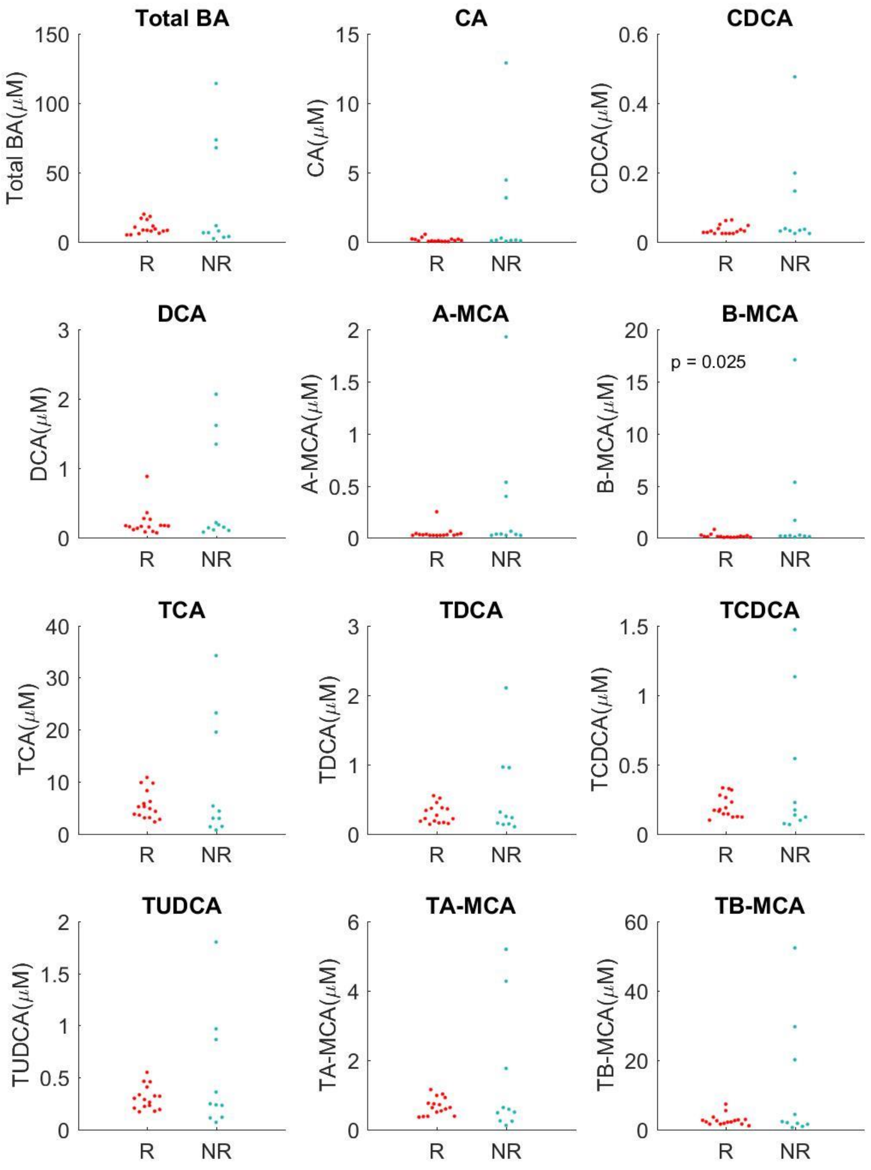

Figure S14

Total plasma bile acids (TBA), cholic acid (CA), chenodeoxycholic acid (CDCA), deoxycholic acid (DCA), alpha-muricholic acid (A-MCA), beta-muricholic acid (B-MCA), tauro-cholic acid (TCA), tauro-deoxycholic acid (TDCA), tauro-chenodeoxycholic acid (TCDCA), tauro-ursodeoxycholic acid (TUDCA), tauro-alpha-muricholic acid (TA-MCA), tauro-beta-muricholic acid (TB-MCA) for all mice in the longitudinal cohort followed for up to 6 months on HFCD. Whenever the difference between R and NR-groups was statistically significant (αϴ.05) using the Wilcoxon rank-sum test, p-values are shown.

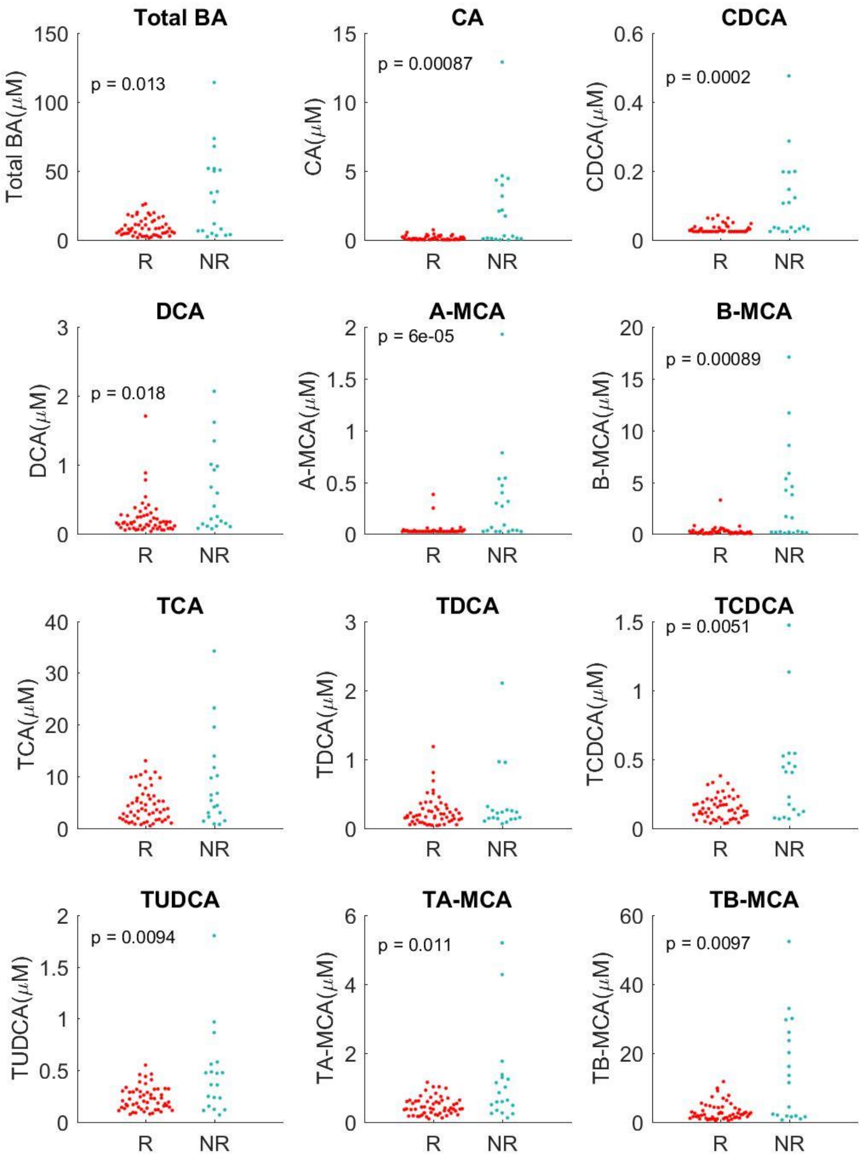

Figure S15

Total plasma bile acids (TBA), cholic acid (CA), chenodeoxycholic acid (CDCA), deoxycholic acid (DCA), alpha-muricholic acid (A-MCA), beta-muricholic acid (B-MCA), tauro-cholic acid (TCA), tauro-deoxycholic acid (TDCA), tauro-chenodeoxycholic acid (TCDCA), tauro-ursodeoxycholic acid (TUDCA), tauro-alpha-muricholic acid (TA-MCA), tauro-beta-muricholic acid (TB-MCA) for all animals used for measurement of fractional cholesterol absorption where mice were fed HFCD for 2 months. Whenever the difference between R and NR-groups was statistically significant ($\alpha \leq 0.05$) using the Wilcoxon rank-sum test, p-values are shown.

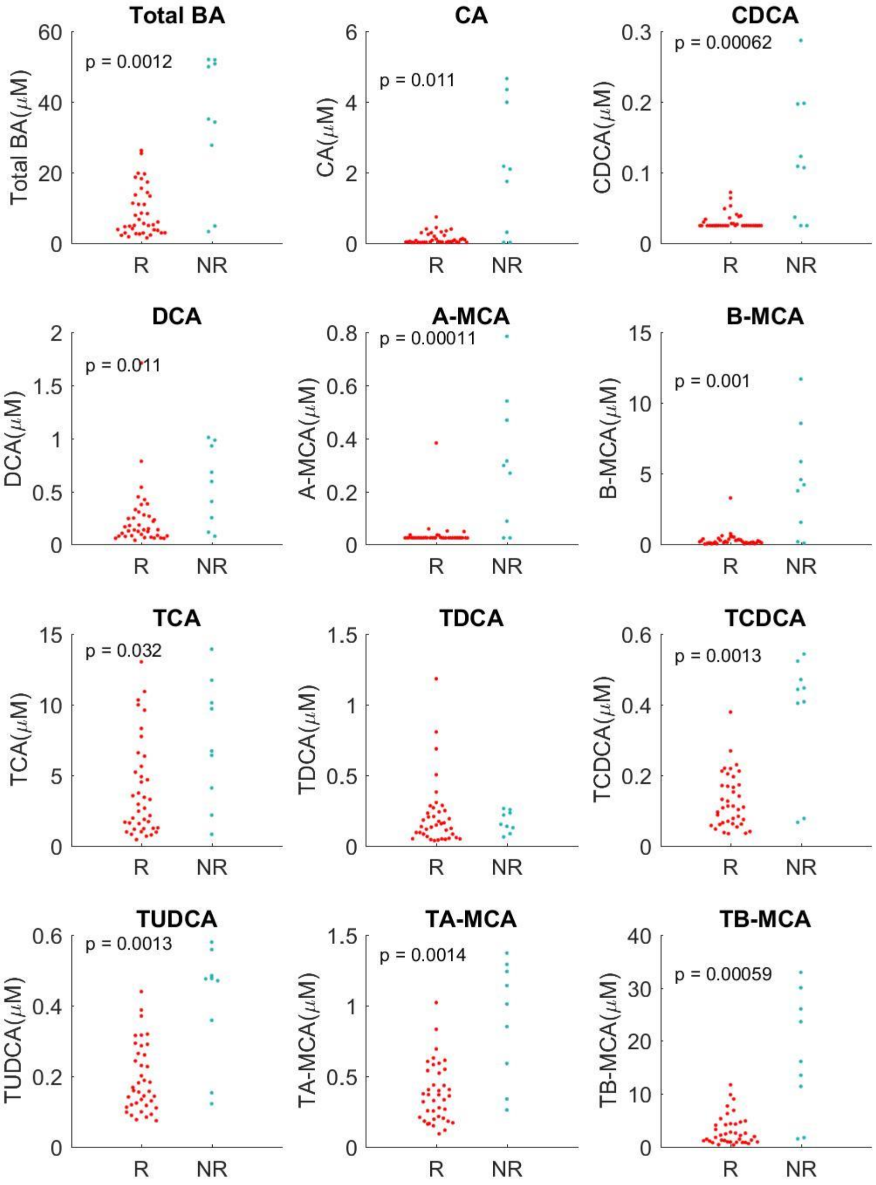

Figure S16

Total plasma bile acids (TBA), cholic acid (CA), chenodeoxycholic acid (CDCA), deoxycholic acid (DCA), alpha-muricholic acid (A-MCA), beta-muricholic acid (B-MCA), tauro-cholic acid (TCA), tauro-deoxycholic acid (TDCA), tauro-chenodeoxycholic acid (TCDCA), tauro-ursodeoxycholic acid (TUDCA), tauro-alpha-muricholic acid (TA-MCA), tauro-beta-muricholic acid (TB-MCA) for all animals used in the longitudinal (6 moths) cohort and validation study together. Whenever the difference between R and NR-groups was statistically significant ($\alpha = 0.05$) using the Wilcoxon rank-sum test, p-values are shown.
